# Editorial: Structure, isotypes, targets, and post-translational modifications of immunoglobulins and their role in infection, inflammation and autoimmunity, Volume II

**DOI:** 10.3389/fimmu.2022.1041613

**Published:** 2022-10-17

**Authors:** Jan Novak, Laureline Berthelot, Sylvie Hermouet

**Affiliations:** ^1^ Department of Microbiology, University of Alabama at Birmingham, Birmingham, AL, United States; ^2^ INSERM, Center for Research in Transplantation and Translational Immunology, UMR 1064, Nantes Université, Nantes, France; ^3^ INSERM, Immunology and New Concepts in ImmunoTherapy, INCIT, UMR 1302, Nantes Université, Nantes, France; ^4^ Laboratoire d’Hématologie, Centre Hospital Universitaire (CHU) Nantes, Nantes, France

**Keywords:** Immunoglobulins, structure, DNA rearrangement, infection, autoimmunity

Post-translational modifications, isotypes, and the antigen/epitope specificity of immunoglobulins are shaped by inflammatory processes and impacted by the genetic background of the host (1–8). These factors contribute to how infection, autoimmunity, and cancer are resolved or, conversely, how autoimmune processes and cancer may progress. The humoral responses, i.e., antibody production, is influenced by the metabolism of plasma cells but also by the isotype of immunoglobulins, composition of their variable segments (variable heavy-chain and variable light-chain sequences), and post-translational modifications (predominantly glycosylation). The activities of antibodies further depend on the expression of corresponding receptors on various cell types. These receptors are Fc receptors (specific for Fc portions of IgG, IgA, IgM, or IgE) and Fc receptor-like molecules (e.g., receptors specific for IgG immune complexes, IgA immune complexes, polymeric IgA, secretory IgA). Thus, affinity of antibodies to antigens and autoantigens is determined predominantly by the variable regions, avidity is determined by the isotype, whereas the sum of effector functions of the antibodies is shaped by relative amounts of different isotypes, their post-translational modifications, and the corresponding receptors on different cells.

This Research Topic features four papers illustrating several important aspects of the structure and function of immunoglobulins, particularly DNA rearrangements and the modification of antibody responses to evade immune defense.

## DNA rearrangements

In autoimmunity, DNA rearrangements seem to restrain the IgM repertoire in individuals with anti-phospholipid syndrome (APS). APS auto-antibodies are reactive with the phospholipid-binding protein β2GPI, among other targets (9–11). Consequently, thrombosis, particularly during pregnancy, is the main complication of APS, and affected patients are typically diagnosed after an abortion. The paper by Pashova et al. describes the IgM repertoire of women with APS and abortions compared to age-matched healthy pregnant women. Pooled Igomes were also compared to the global public repertoire Igome of donor IgM and 7-mer sequences found in the J regions of human immunoglobulins. The IgM repertoire of the APS patients exhibited a significant reduction of certain public specificities found in healthy controls with targets representing low complexity linear self-epitopes homologous to human antibody J regions.

DNA rearrangement and maturation of immunoglobulins are an important phase of antibody response. In their paper “*Tandem Substitutions in Somatic Hypermutation*”, Sepúlveda-Yáñez et al. examined the incidence and mechanism of tandem substitutions in human peripheral blood B cells. The authors show that affinity maturation of the B-cell receptor through somatic hypermutation includes substitutions (especially tandem dinucleotide substitutions) and postulate that the process involves uracil DNA glycosylase. These tandem substitutions enhance affinity maturation during the adaptive immune responses by enabling mutations of two adjacent amino-acid residues at the same time. This ubiquitous presence of tandem substitutions in human V(D)J rearrangements can overcome amino-acid codon degeneracy.

## Modification of antibody responses to evade immune defense

During infection, both virus and bacteria seem to modify antibody responses to evade immune defenses. In “*Monomeric IgA Antagonizes IgG-Mediated Enhancement of DENV Infection*”, Wegman et al. show on the example of antibodies against dengue virus that some IgG antibodies may exhibit infection-enhancing activity, i.e., antibody-dependent enhancement. As there are four serotypes of dengue virus, prior infection with one dengue virus serotype may induce IgG antibodies weekly binding to another dengue virus serotype. Such IgG antibodies may exhibit infection-enhancing activity. The authors showed that dengue-virus-specific IgA antibodies do not have such infection-enhancing activity but instead exhibit antagonizing effects against infection-enhancing IgG. These experiments thus indicate that more comprehensive information about the patterns and titers of antibody responses may provide a better predictor for disease risk. To understand the mechanism behind IgA/IgG antagonizing, further studies on receptor engagement and cellular signaling are needed. However, IgA can inhibit myeloid cells by binding to their Fcα receptors (FcαRI) and potentially block IgG-induced signaling (12).

Regarding Lyme disease, the paper by Haslund-Gourley et al. (“*Acute Lyme disease IgG N-linked glycans contrast the canonical inflammatory signature*”), characterizes the N-glycosylation of IgG by mass spectrometry and discovers glycan patterns that discriminate acute Lyme disease patients from healthy controls and treated patients. Acute Lyme disease was characterized by a general increase of galactosylation of IgG N-glycans. By contrast, in the context of inflammation, IgG has usually reduced galactose content (Dekker et al., 2017). In this particular infection by *Borrelia*, IgG glycosylation is altered, suggesting a global subverted immune response. Moreover, galactosylation of IgG can directly modify the binding of IgG to some Fc receptors and complement C1q, thus impacting antibody-dependent cellular cytotoxicity (ADCC) and antibody-dependent cellular phagocytosis (ADCP) (13–15). Importantly, Haslund-Gourley et al. discuss several mechanisms that potentially impact IgG N-glycosylation during Lyme disease: i) a changed cytokine micro-environment that alters the expression of the glycosyltransferase in B cells and/or plasma cells; ii) the destruction of germinal centers; iii) infection of endothelial cells; iv) alteration of the N-glycome due to changes in gut flora following doxycycline therapy.

Altogether, both infections, modifying isotype of antibody response or glycosylation could impact cellular responses *via* modifications of Fc region and binding to Fc receptors.

## Conclusion

This Research Topic brings new information on immunoglobulin structures in the contexts of infection and auto-immunity, highlighting the complexity of their regulation. Both DNA rearrangements and post-translational modifications impact immunoglobulin functions.

## Author contributions

All authors have made a direct intellectual contribution to the editorial and approved it for publication.

## Conflict of interest

The authors declare that the research was conducted in the absence of any commercial or financial relationships that could be construed as a potential conflict of interest.

## Publisher’s note

All claims expressed in this article are solely those of the authors and do not necessarily represent those of their affiliated organizations, or those of the publisher, the editors and the reviewers. Any product that may be evaluated in this article, or claim that may be made by its manufacturer, is not guaranteed or endorsed by the publisher.
